# Teaching the National Institutes of Health Stroke Scale to Paramedics (E-Learning vs Video): Randomized Controlled Trial

**DOI:** 10.2196/18358

**Published:** 2020-06-09

**Authors:** Avinash Koka, Laurent Suppan, Philippe Cottet, Emmanuel Carrera, Loric Stuby, Mélanie Suppan

**Affiliations:** 1 Division of Emergency Medicine Department of Anaesthesiology, Clinical Pharmacology, Intensive Care and Emergency Medicine Geneva University Hospitals Geneva Switzerland; 2 Stroke Center Department of Neurology Geneva University Hospitals Geneva Switzerland; 3 Genève TEAM Ambulances Geneva Switzerland; 4 Division of Anesthesiology Department of Anaesthesiology, Clinical Pharmacology, Intensive Care and Emergency Medicine Geneva University Hospitals Geneva Switzerland

**Keywords:** active learning, electronic learning, video, stroke, online learning, e-learning

## Abstract

**Background:**

Prompt and accurate identification of stroke victims is essential to reduce time from symptom onset to adequate treatment and to improve neurological outcomes. Most neurologists evaluate the extent of neurological deficit according to the National Institutes of Health Stroke Scale (NIHSS), but the use of this scale by paramedics, the first healthcare providers to usually take care of stroke victims, has proven unreliable. This might be, at least in part, due to the teaching method. The video used to teach NIHSS lacks interactivity, while more engaging electronic learning (e-learning) methods might improve knowledge acquisition.

**Objective:**

This study was designed to evaluate whether a highly interactive e-learning module could enhance NIHSS knowledge acquisition in paramedics.

**Methods:**

A randomized controlled trial comparing a specially designed e-learning module with the original NIHSS video was performed with paramedics working in Geneva, Switzerland. A registration number was not required as our study does not come into the scope of the Swiss federal law on human research. The protocol was nevertheless submitted to the local ethics committee (Project ID 2017-00847), which issued a “Declaration of no objection.” Paramedics were excluded if they had prior knowledge of or previous training in the NIHSS, or if they had worked in a neurology or neurosurgery ward. The primary outcome was overall performance in the study quiz, which contained 50 questions. Secondary outcomes were performance by NIHSS item, time to course and quiz completion, user satisfaction regarding the learning method, user perception of the course duration, and probability the user would recommend the course to a colleague.

**Results:**

The study was completed by 39 paramedics. There was a better overall median score (36/50 vs 33/50, *P*=.04) and a higher degree of satisfaction regarding the learning method in the e-learning group (90% vs 37%, *P*=.002). Users who had followed the e-learning module were more likely to recommend the course to a colleague (95% vs 63%, *P*=.02). Paramedics in the e-learning group took more time to complete the course (93 vs 59 minutes, *P*<.001), but considered the duration to be more adequate (75% vs 32%, *P*=.01). Time to quiz completion was similar between groups (25 vs 38 minutes, *P*=.12).

**Conclusions:**

Use of an e-learning module shows promising results in teaching the NIHSS to paramedics.

## Introduction

### Background and Importance

Prompt and accurate identification of stroke victims is essential to reduce time from symptom onset to adequate treatment and to improve neurological outcomes [1]. Evaluation of the extent of the neurological deficit is generally performed according to the National Institutes of Health Stroke Scale (NIHSS), which was first described in 1989 [2,3]. Although the NIHSS has been extensively studied and validated in the hospital setting for many different types of provider, data regarding its application in the prehospital setting is limited [4]. Moreover, in contrast to other stroke scales, use of the NIHSS by Helicopter Emergency Medical Services crews has proved unreliable [5-8]. Nevertheless, the NIHSS enables identification of neurological deficits often missed when using other scales, particularly in cases of cerebellar stroke [9]. Accurate and reliable use of the same scale as that used by hospital neurologists might enable paramedics to improve the prehospital triage of stroke patients, and could help decrease door-to-needle time in patients for whom thrombolysis can be considered [10].

Many different factors might contribute to the difficulty of using the NIHSS in acute stroke, including the method used to teach this scale to paramedics. The video developed by Patrick Lyden et al is the most studied method used to teach NIHSS to in-hospital providers [11]. This media has many advantages, as it does not require the presence of a teacher or of a patient, and can be used at any time, even during night shifts. Lack of interactivity, however, has also been noted as a potential shortcoming [4].

Electronic-learning (e-learning) in its various forms is increasingly used in health professional education. Highly interactive self-paced modules are part of current e-learning models and often elicit higher satisfaction in the learning population [12,13]. Greater satisfaction with the learning method is usually associated with better understanding and performance [14].

### Goal of This Investigation

The aim of our study was to evaluate the performance of an e-learning module in teaching the NIHSS to paramedics. Our hypothesis was that an e-learning module should, through its higher degree of interactivity, increase the paramedics’ performance.

## Methods

### Study Design and Setting

This was a randomized (1:1), controlled, outcome assessor and data analysts blinded superiority study compliant with the CONSORT-EHEALTH guidelines [15]. The study took place in July 2019 in Geneva, Switzerland. In this state, paramedics need to follow a 3-year curriculum before certification, during which they are taught to assess potential stroke patients using simplified scales such as the Cincinnati and G-FAST, but not the NIHSS. Geneva emergency dispatchers send an ambulance staffed with two certified paramedics whenever they identify a possible stroke. Medical reinforcement by a physician-staffed mobile unit (Service Mobile d’Urgence et de Réanimation – SMUR) is only sent if an immediate vital threat is identified by the dispatchers or ambulance crew [16].

The study protocol (Project ID 2017-00847) was submitted to the local ethics committee, which issued a “Declaration of no objection” as this project does not come into the scope of the Swiss federal law on human research. A registration number was therefore not required.

### Subjects and Enrollment

All paramedics working for any of the 7 ambulance services in Geneva and/or in the SMUR unit were invited to join the study on a voluntary basis. Four training sessions were organized on two different days to ensure maximum participation despite the shift work pattern. Paramedics were excluded if they had prior knowledge of or previous training in the NIHSS, or if they had worked in a neurology or neurosurgery ward. Consent was obtained through the online platform.

### Learning Material and Online Platform

An e-learning module containing 184 highly interactive slides was created by 4 of the authors (MS, LSt, LSu, and AK) under Storyline 3 (Articulate Global). The entire module is available online at https://e-learning.nihss-study.ch. To make navigation easier and to allow the end user to easily review the learning material, this module is divided into chapters, each representing one item on the NIHSS. Each chapter is composed of 3 parts: an initial description explaining the scoring of the item, an illustration of how to test the patient, and a summary of the scoring and key pitfalls to look out for. To increase interactivity and user involvement, challenging questions are presented to the participants at the beginning of each chapter prior to displaying key learning material to stimulate curiosity [17]. Each wrong answer leads to a cue or to a detailed explanation before the user can either retry or move forward. Completion of all the chapters enables access to a practice quiz covering all scoring items.

Parts of the original video by Lyden et al are embedded in the e-learning module to illustrate clinical testing, with permission of the author. The original video had to be subtitled as English language skills vary widely among the paramedics working in Geneva. It was therefore fully translated by one author (MS), who also created the required subtitles. Three of the other authors (AK, LSt, LSu) reviewed the subtitled video to ensure the quality and accuracy of the translation. The subtitled video is available at https://video.nihss-study.ch.

The learning material was uploaded to an online platform developed under the Joomla! 3.9 Content Management System (Open Source Matters).

### Study Sequence

Once paramedics had registered for the study, one investigator (LSt) sent the total number of registered participants to a second investigator (MS). This second investigator, thus blinded to the participants’ information, performed randomization with a 1:1 allocation using a computer-generated table (https://www.randomizer.org/), and created unique identifiers (usernames and passwords). These identifiers, that would allow paramedics to log into the e-learning platform, were printed and placed in identical, opaque, sealed envelopes. These envelopes were shuffled and randomly scattered on a table by one of the co-authors (LSt or LSu) before each study session. Paramedics were asked to randomly choose one of the envelopes upon entering the study room, and to sit in front of a computer. Paramedics discovered their allocated group (e-learning vs video) only after logging into the online platform. They were then asked a set of questions designed to gather demographic data and to screen for exclusion criteria before being able to access the learning material.

The control group watched the subtitled video with unrestricted access to the controls, allowing them to pause, rewind, or move forward as they wished. The experiment group was given access to the e-learning module and could freely review and navigate the e-learning chapters at will. Each group only had access to their learning media, and no time constraints were imposed.

All participants could take notes while consulting the learning material and use them during the study quiz but were not provided with a NIHSS form. For both groups, access to the study quiz was enabled only after completion of the learning material. The study quiz was identical for all participants and contained 50 questions. The first 5 questions were related to key NIHSS concepts and were followed by full NIHSS evaluations of three patients. These evaluations were taken from the Lyden certification videos and were displayed according to the regular NIHSS scoring logic. To allow the candidates to immediately score the item they had viewed, each evaluation was divided into 15 subtitled video extracts, which could be paused, forwarded, or rewound at will. None of these extracts were shown to the paramedics either in the control video or in the e-learning module.

Once the quiz was completed, a last set of questions, based on a 5-point Likert scale, was displayed in order to assess the paramedics’ feelings and thoughts regarding the length of the course, their satisfaction regarding the teaching method, the perceived difficulty, and the probability they would recommend the course to a colleague.

### Data Collection

Data was electronically and automatically recorded in a MariaDB database version 5.5.5 (MariaDB Foundation) before being extracted to an Excel spreadsheet (Microsoft Corporation). Group-related data was coded before the spreadsheet was sent for data analysis to the same investigator who performed the randomization (MS) in order to ensure the blinding.

The original data have been deposited to Mendeley Data [18].

### Outcomes

The primary outcome was overall performance in the study quiz. Secondary outcomes were performance by NIHSS item, time to course and to quiz completion, user satisfaction with the learning method, user perception of the duration of the course, and probability that the user would recommend the training to a colleague.

### Statistical Analysis

Based on prior teaching experience and clinical assumption, we determined that a sample size of 34 (17 per group) was required to have an 80% chance of detecting a difference of 10% in the overall performance at the 5% level of significance.

Given the low sample size, we decided to use only non-parametric statistical tests. The Fisher exact test was used for categorical variables and the Mann-Whitney U test for continuous variables. A two-sided *P-*value <.05 was considered significant.

Data analysis was performed using STATA 16.0 (Stata Corporation).

## Results

### Characteristics of Study Subjects

Of the 40 paramedics who registered to one of the four study sessions, 39 (98%) completed the study, none of whom met any of the exclusion criteria. One paramedic who was expected to participate did not appear for the study session. Gender distribution was the only statistically significant variable between groups (2/19 women in the e-learning group vs 10/20 in the video group, *P*=.01) (Table 1).

**Table 1:** Participant characteristics.

Characteristic		Video (n=19)	E-learning (n=20)	*P* value
**Age range (years), n**				.46
	18-25	2	1	
	26-30	3	7	
	31-35	6	6	
	36-40	4	3	
	≥41	4	3	
Years since diploma, median (Q1;Q3)		5 (2;8)	6.5 (2.5;9)	.68
Gender, female, n (%)		2 (11)	10 (50)	.01

### Main Results

Participants who followed the e-learning module performed better than those who followed the video—36 (34;37) vs 33 (31;38), *P*=.04. Paramedics who had followed the e-learning module had better scores regarding key NIHSS concepts (*P*=.01), the consciousness—commands item (*P*=.03), and the ataxia item (*P*=.02). Paramedics in the video group evaluated dysarthria better than those in the e-learning group (*P*=.02). Detailed results are shown in Table 2.

**Figure 1:**
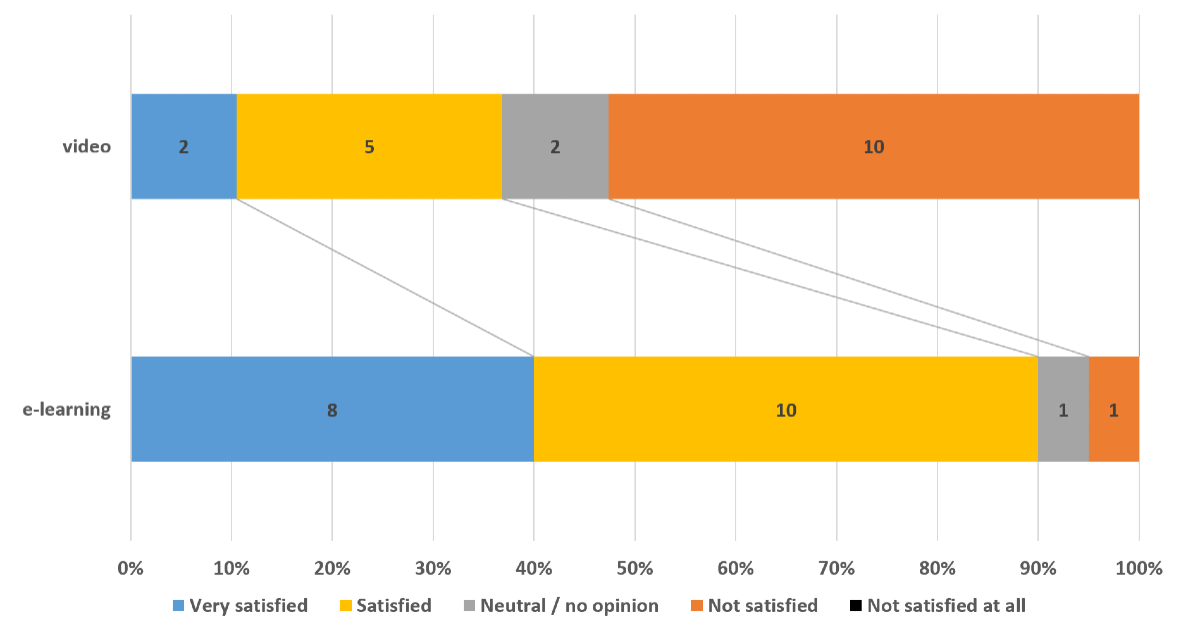
Satisfaction by group.

The e-learning module took more time to complete than the video with a median time of 93 (81.5;107.5) vs 59 (55;80) minutes, *P*<.001, though time to quiz completion was not significantly different between groups (*P*=.12) (Table 2). Duration of the course was considered adequate by 75% of the paramedics who had followed the e-learning module, but only by 32% of those who had followed the video (*P*=.01). Satisfaction regarding the learning method was also higher in the e-learning group compared to the video group (90% vs 37%, *P*=.002) (Figure 1). The paramedics who had followed the e-learning module were more likely to recommend the course than those who had followed the subtitled video (95% vs 63%, *P*=.02).

**Table 2:** Quiz results. Data are expressed as median (Q1;Q3).

Variable		Video (n=19)	E-learning (n=20)	*P* value
Overall score		33 (31;38)	36 (34;37)	.04
Time to course completion (min)		59 (55;80)	93 (81.5;107.5)	<.001
Time to quiz completion (min)		38 (33;41)	35 (31;39)	.12
**Detailed results by item**				
	Key NIHSS^a^ concepts	5 (3;5)	5 (5;5)	.01
	Consciousness–Global	2 (2;2)	2 (2;3)	.30
	Consciousness–Questions	3 (2;3)	3 (2;3)	.40
	Consciousness–Commands	2 (2;2)	3 (2;3)	.03
	Gaze	3 (2;3)	3 (2;3)	.85
	Visual	2 (2;2)	2 (2;2)	1
	Facial Palsy	1 (0;2)	1 (0;2)	.71
	Motor arm	4 (3;5)	4 (4;5)	.75
	Motor leg	5 (4;6)	5 (4;5)	.69
	Ataxia	1 (0;1)	1 (1;2)	.02
	Sensory	2 (2;3)	3 (2;3)	.40
	Language	1 (1;1)	1 (1;2)	.41
	Dysarthria	2 (2;3)	2 (2;2)	.02
	Extinction and inattention	2 (1;3)	3 (2;3)	.35

^a^NIHSS: National Institutes of Health Stroke Scale.

## Discussion

### Main Results

The participants who followed the e-learning module performed slightly better than those who watched the subtitled version of the original NIHSS video. Our results show that paramedics who followed the e-learning module had a better understanding of the NIHSS key concepts. This difference might prove useful for the application of the NIHSS in the field.

While some specific NIHSS items, such as ataxia, were scored more accurately by the paramedics who followed the e-learning module, participants in the video group evaluated the dysarthria item significantly better. After careful review of the module, it appears that we did not include extracts from the original video for all 15 items. Indeed, the e-learning module used in this study did not contain any video extract demonstrating how to assess dysarthria. This shortcoming might, at least in part, explain why the paramedics who followed the e-learning module did not perform as well as those who had seen the video. The e-learning module should therefore be updated to include video extracts of each specific item before being tested again to assess whether our hypothesis holds true. The difference observed regarding dysarthria evaluation might also be explained by the fact that this item has been shown to have a low interrater reliability [19].

The e-learning module took longer than the video to complete, with a median difference of 34 minutes. Nevertheless, 75% of the paramedics who had followed the e-learning module judged its length adequate, whereas less than a third of those who had followed the video were of the same opinion. Three main factors might contribute to this finding. First, while the video does not allow for interactions other than pausing, forwarding, or rewinding, the e-learning module stimulates and engages the learners. Second, the possibility of quickly going back to the learning chapter one wishes to review is facilitated by the e-learning interface, as the learner loses less time looking for the right sequence. Last, the introductory chapter in the e-learning module seems to lead to an enhanced understanding of key NIHSS concepts, and might therefore help the learner better understand the goals of the course, therefore acting as a primer by increasing both the learner’s commitment and attention.

The higher level of satisfaction with the learning method is probably most strongly linked to the high interactivity of the e-learning module. Previous studies have indeed shown that a greater level of interactivity increases learner engagement while decreasing the attrition rate [20]. Though other factors such as quality of content, convenience of the technology, and quality of the support received have also been found to increase learner satisfaction [21], they probably had little influence on this outcome given our study design as these elements were almost identical for both groups.

The term e-learning is generic and is used to describe any kind of educational material made available through technological means. It therefore covers many different formats (video, podcast, interactive module, serious game, etc), which have been increasingly used in the last decades because of their many advantages such as flexibility, mobility, and avoidance of both time and space constraints [22]. Advanced models of e-learning can mimic real-life situations and are often considered preferable to classical models of education thanks to their interactivity. Systematic reviews have however failed to demonstrate a superiority of e-learning compared to traditional learning in improving patient outcomes or health professionals’ behaviors, skills, or knowledge [23,24]. This might be because e-learning materials are so different in conception and philosophy that they cannot be directly comparable.

Even if it cannot entirely substitute for face-to-face learning, e-learning seems to be particularly well adapted to the field of emergency medicine where shift work is common [25]. It is indeed often difficult to provide recurrent practical hands-on sessions for paramedics once they have completed their training, though they must still acquire new knowledge and skills on a regular basis. Studies have shown that e-learning is the preferred learning method for paramedics when practical training is not possible due to diverse constraints [26,27].

Stroke is the second leading cause of death worldwide and a major cause of long-term disability [28]. One of the best ways to limit stroke sequelae is to reduce the time between symptom onset and administration of the appropriate treatment. Swift and accurate identification of stroke symptoms is therefore of paramount importance to enable prompt transport to a stroke center. Some studies suggest that the use of e-learning material enhanced both neurological assessment by nurses and quality of care in stroke teams [29,30]. Different e-learning tools aiming to improve symptoms recognition and global care of stroke patients are currently being developed and implemented [31,32].

### Strengths and Limitations

Though this study has several strengths, it also has limitations. First, given our initial power calculation and the small number of paramedics working in Geneva, the sample size is limited. Moreover, as there can be considerable differences between prehospital emergency medical systems or paramedic certifications, even across different Swiss regions, the results of this study might not be entirely applicable to other systems [16]. As this study only assessed learning material, it also might not adequately predict the performance of the paramedics in the field. Finally, as the video had a fixed duration of about 59 minutes, the main investigator might have been only incompletely blinded as to group allocation. Nevertheless, randomization, concealment, and blinding were performed according to the strictest standards. Comparison of this innovative highly interactive e-learning module with a well-established teaching standard is another strength of this study.

### Implications for Future Research

After an update and improvement of the e-learning module used in this study, a new assessment of this teaching method should be performed. The performance of healthcare providers trained with this new method and its impact on the global care of stroke patients should also now be assessed in the field.

### Conclusion

Use of an e-learning module shows promising results in teaching the NIHSS to paramedics. The module should be improved according to the study results and user feedback, and performance of healthcare providers who have followed this module should be tested in the field.

## Appendix

Multimedia Appendix 1 CONSORT-eHEALTH checklist (V 1.6.1).

## Abbreviations

NIHSS: National Institutes of Health Stroke Scale
SMUR: Service Mobile d’Urgences et de Réanimation
